# Development and Psychometric Assessment of the Consumer Socio-Cultural Premises Scale in Mexico

**DOI:** 10.11621/pir.2025.0202

**Published:** 2025-05-01

**Authors:** Francisco Leonardo Soler-Anguiano, Rolando Díaz-Loving, Alejandra del Carmen Domínguez-Espinosa

**Affiliations:** a National Autonomous University of Mexico, Mexico City, Mexico; b Iberoamerican University, Mexico City, Mexico

**Keywords:** social norms, sociocultural, scale development, consumer behaviour

## Abstract

**Background:**

Investigating the complexities of consumer behaviour requires an understanding of the sociocultural contexts that shape individual preferences and purchasing patterns. Factors such as family dynamics, community norms, and cultural values emphasise the importance of exploring the influence of sociocultural premises. These forces are continuously negotiated within the evolving trends of the global consumer culture. As a result, it is essential to recognise that each group has unique values and needs that must be acknowledged.

**Objective:**

This study aims to explore, develop, and assess psychometric properties and evidence of a measure assessing consumer sociocultural premises from an ethnopsychology approach, to identify distinctive elements shaping purchasing patterns based on social norms.

**Design:**

A mixed-methods research design was employed to gather qualitative and quantitative data for a comprehensive analysis. First, sociocultural norms were extracted through focus group discussions. Building upon these identified premises, the subsequent study developed a scale wherein scale items were created, and their psychometric properties were evaluated through exploratory and confirmatory factor analyses.

**Results:**

The first study identified sociocultural premises related to family, gender roles, self-sacrifice, product, and hedonism that shape consumer behaviour. In the second study, these elements were incorporated into the initial set of scale items, which were then refined through expert review, pilot testing, and statistical analyses. Results revealed a seven-factor structure reflecting the key socio-cultural premises identified in the focus groups. The scale demonstrated strong internal consistency and adequate psychometric properties evidence.

**Conclusion:**

The findings of this study underscore the significance of sociocultural factors in shaping individual purchasing behaviour, emphasising the need for a measurement tool that captures culturally specific purchasing beliefs and universal factors. This research contributes to the existing body of knowledge on social dynamics and consumer behaviour, providing valuable insights for future studies and practical applications in the field.

## Introduction

In our daily lives, we’ve all had to wait in line, whether at the grocery store, the bank, or when buying tickets for an event. The idea of “first come, first served” is usually followed, reflecting the social norm of fairness, with consequences for those who don’t follow it. These consequences range from mild annoyance to severe reactions (Helweg-Larsen & Lomonaco, 2008). In Mexico, culturally accepted ways of breaking this norm have been established, as seen in the popular saying “he who went to La Villa lost his chair,” which refers to the risk of losing one’s place in a queue due to carelessness. This suggests that verbal expressions and cultural norms have significant influence beyond their literal meaning (Fagundes, 2017).

Life in society necessitates a collective consensus on various aspects. Asch (1955), Cialdini and Trost (1988), and Sherif (1936) demonstrated this by their findings in their studies on conformity. Individuals often adhere to social norms, a process that shapes their behaviour and even self-identity (Tajfel, 1978). Building on the social identity theory, (which posits that individuals derive their sense of self and belonging from the social groups they identify with), we propose that consumer behaviour is significantly shaped by the socio-cultural premises or core beliefs that are salient within these reference groups. This is particularly evident in consumer behaviour, where factors such as culture, personal values, and social interactions significantly influence purchasing preferences (Ribeiro et al., 2021). Some studies have explored how cultural values serve as guidelines that shape group behaviours, thoughts, and feelings (Rahman et al., 2021). Venkatesan’s (1966) research suggests that individuals frequently follow social norms in purchasing decisions so as to align with societal expectations. These characteristics can be viewed as a collective mental programming of groups, akin to Hofstede’s (2001) conceptualisation of culture — which is distinctions in actions, feelings, and thoughts within groups. Therefore, culture provides the framework and environment that shapes behaviours and decisions. Thus, culture furnishes the structure and environment in which behaviours and decisions are shaped.

When cultural elements are incorporated into models of diverse behaviours, research has highlighted the more indirect nature of these influences. This is reflected in models developed by Betancourt et al. (2010), which identify three key areas within healthy behaviour. The first involves the psychological processes and personal aspects, such as individual-level emotions, which have the closest and most outstanding impact on behaviour. The second relates to cultural elements, including value orientations, beliefs, and norms that are socially shared within a population, which can directly or indirectly influence behaviour through psychological processes. The third area consists of the broader population categories, which are the most distal elements and may not necessarily be associated with a particular behaviour. According to the Flynn et al. (2011) model, structural social factors are more likely to be associated with variations in cultural aspects rather than directly with behaviour. In consumer domains, the influence of social and cultural variables has been integrated into models as important predictors and moderators. These variables have been shown to impact various scenarios, including environmentally responsible purchases (Thøgersen, 1999), purchase intentions (Moon et al., 2008), and intertemporal preferences (Appelhans et al., 2019). The challenge is to identify culturally specific elements of Mexican culture that influence purchasing products and services, considering implicit rules and subjective formations that shape the perceptions of individuals. This endeavour holds theoretical significance for the study of sociocultural influences across different domains and offers valuable insights into consumer behaviour with cultural relevance.

Based on the historical-bio-psycho-socio-cultural theory (Díaz-Guerrero, 1972) there is a proposition that behaviour can be understood from the social and cultural analysis in which individuals are immersed, based on the norms and premises that provide structure and govern the behaviour of individuals. The way to unify the effect of culture with behaviour, according to Díaz-Guerrero (1972), is by integrating psychological components with a bioevolutionary perspective and socio-cultural variables that provide the structure of behaviours. Thus, from this perspective, individuals develop their personal attributes derived from the dialectic between biopsychic needs and norms, premises, and values o f the group to which the individual belongs. These historical-socio-cultural premises, initially linked to assertions about the Mexican family, have been further explored in specific contexts such as monogamy (Escobar-Mota & Sánchez-Aragón, 2013), emotional expression (Sánchez-Aragón & Díaz-Loving, 2009), gender stereotypes (Rocha-Sánchez & Díaz-Loving, 2005), and Mexican university students (Cruz et al., 2009). This indicates the presence of identifiable cultural elements across various realms of individual interaction. Could similar premises extend to economic domains? The presence of cultural propositions such as the song “Can’t Buy My Love” or the Mexican saying “A paid mariachi band plays a bad song” could suggest elements governing individual behaviour in economic transactions and consumption.

Existing research has highlighted the significant influence of cultural factors on consumption patterns (Rong et al., 2020). However, there is a need to consider not only the cultural factors affecting consumer behaviour but also the possible variations among groups (Deshpandé et al., 1986; McCort & Malhotra, 1993; Rahman et al., 2021; Ribeiro et al., 2021). There remains a gap in investigating the heterogeneity of these cultural influences across diverse contexts. Examining consumption within different subcultures could yield novel and beneficial findings to implement effective social programmes and marketing strategies (Deshpandé et al., 1986; McCort & Malhotra, 1993).

To enhance the understanding of consumer behaviour, it is essential to identify the key cultural components within a specific population and develop a measure to model the effects of these cultural premises on consumer behaviour. This research endeavours to address the existing gap by focusing on the examination of the premises that impact consumer behaviour amongst Mexican consumers and the construction of a scale to quantify these influential factors. The study posits that culture, as manifested through socio-cultural premises, plays a pivotal role in shaping consumer behaviour, through the identification and measurement of these premises.

## Study 1

### Methods

The principal aim of the study is to investigate the prevalent norms and beliefs surrounding purchasing behaviours. To achieve this, an exploratory approach was adopted using a qualitative narrative design and the focus group technique to gather data. An interview guide with a semi-structured format has been developed to facilitate natural discussions among participants regarding their purchasing habits, norms, and beliefs.

#### Participants

Thirty-four participants from Mexico City (20 women and 14 men), aged 18 to 65 with a mean age of 35 years (SD = 16.42), were recruited using convenience non-random sampling. The educational background of the participants varied, with 58.8% having a maximum of high school education, 24.4% with university studies, 8.8% with high school studies, and the remaining participants having primary school education. To be eligible for the study, participants had to be of legal age, employed in a remunerated job for at least one year, and have experience in that job or activity. The study also considered the socioeconomic status of the participants, which was categorised according to the Mexican Association of Market Research Agencies’ classifications (AMAI, 2024). The sample included 15.6% of participants in level A/B, 31.3% in level C+, 25% in level C, 25% in level C-, and 3.1% in level D+.

All individuals were invited to participate voluntarily without receiving any financial compensation. Their data’s anonymity and confidentiality were assured, and consent was obtained to record the session. The informed consent for the session and the recording were provided verbally and in writing, and participants provided their signatures to indicate their agreement. The study and consent procedures were performed in accordance with the ethical standards of the Declaration of Helsinki (World Medical Association, 2013), and the ethical guidelines of the university committee.

#### Procedure

##### Questionnaires

A discussion guide was developed for the focus groups, centering on broad concepts related to acquiring goods and services. Participants were prompted to discuss their motivations for purchasing, using a hypothetical scenario in which they had $10,000 MX ($598 US) at their disposal. They were also asked whether they would seek advice from others and what input they might receive from family, friends, and acquaintances regarding their spending choices. The discussions delved into the underlying cultural norms and beliefs reflected in common sayings and proverbs related to consumer behaviour. Lastly, participants were invited to share their perspectives on potential differences in shopping behaviours between men and women.

The Socioeconomic Level Questionnaire (AMAI, 2024) was used to assess and categorise Mexican households based on their ability to provide for their members. This was determined by six criteria, including the parents’ education level and the number of bedrooms and bathrooms. Individuals are placed into category A/B if most household heads have professional education, prioritise spending on education, and allocate less to food. Level C+ is for those with at least one vehicle and fixed internet, devoting more to food and transportation. Level C is for households with a head of family with more than primary education and less investment in education. Level C- is for households with fixed internet connection and allocating around half of their income to food, transportation, and communication. D+ level is for households with fixed internet access and spending just under half of their income on food. Level D is for households where the head of the family has up to primary education, with a small proportion having fixed internet access. Level E is for households with minimal internet access at home, spending just over half of their income on food.

##### Data Analysis

The study employed a categories content analysis approach, as described by Krippendorff (1980), to examine the focus group discussions. The recorded sessions were transcribed and analysed, and an inductive method was used to identify meaningful elements and indicators related to purchasing norms, which were then categorised. Frequencies were tallied for each category and group, and chi-square homogeneity tests were conducted to determine any significant differences related to the premises given across women and men.

### Results

461 indicators have been identified and subsequently categorised into nine general dimensions. Examining the focus group findings by biological sex using chi-square tests revealed statistically significant differences in several dimensions (see *Table 1*). Regarding gender stereotypes, women expressed significantly more premises related to the differential roles of men and women in the home economy, the family’s wellbeing, and appropriate spending behaviour. Additionally, women reported more premises about social acceptance and prestige, as well as self-sacrifice in their consumption choices, compared to men. In contrast, men expressed significantly more premises related to product attributes.

**Table 1:** Content Analysis of the Sociocultural Premises of Purchasing

Categories	Definition	Example	Total	Frequencies by sex		*χ^2^*
				Women N = 20	Men N = 14	
Family	The assigned roles for family members during shopping	*“Parents must call the shots on what gets bought for the house”*	125	73 (*58.4%*)	52 (*41.6%*)	.142
Gender stereotype	The gender roles associated with shopping and the societal expectations for men and women in this context	*“When guys go into a store, they usually end up buying the first thing they see”*	**79**	58 (*73.4%*)	21 (*26.6%*)	6.999^**^
Social	The influence of peers, acquaintances, or industry experts on consumer purchasing behaviour	*“If someone already knows about the product, they know more than you do”*	**58**	30 (*51.7%*)	28 (48.*3%*)	1.777
Saving & Budgeting	Prioritising the economy’s well-being and emphasising the necessity of making purchases based on needs	*“When you’re buying, you just gotta make do with what you’ve got”*	**49**	29 (*59.2%*)	20 (*40.8%*)	.010
Product	Product attributes encompassing quality, functionality, and price	*“Just because something’s cheap doesn’t mean it’s worse”*	**45**	16 (*35.6%*)	29 (*64.4%*)	11.999^***^
Social Acceptance and Status	Acquiring products with the intention of showcasing social status or securing approval within a specific social circle	*“You buy expensive stuff because it’s the image you show to others”*	**40**	30 (*75%*)	10 (*25%*)	4.099^*^
Self-sacrifice	Striving harder and enduring hardship to acquire necessary purchases	“To get what you want, you have to figure out how to get the money to do it”	**25**	21 (*84%*)	4 (*16%*)	5.330^**(a)^
Hedonism	The pursuit of pleasure as an incentive for consumer purchasing decisions	*“We tend to buy things because we want them, not because we need them”*	**25**	13 (*52%*)	12 (*48%*)	.374^(a)^
Affective compensation	Purchase meaningful gifts for loved ones as a way to express affection and replace time spent together	*“You wanna get your son a treat to keep him motivated”*	**15**	12 (*80%*)	3 (*20%*)	^(b)^

*p < .05, **p < .01, ***p < .001 *(a) Yates Correction, (b) Fisher’s Exact Test*

## Study 2

### Method

#### Participants

The study involved 309 participants (53.7% women and 46.3% men) who met specific eligibility criteria, including being employed and at least 18 years old. The participants were chosen using a convenience non-random sampling method and ranged in age from 18 to 67 years (with a mean age of 32.95 and a standard deviation of 13.55). Regarding educational attainment, 61.5% held bachelor’s degrees, 24.9% completed high school, 6.8% pursued postgraduate studies, 6.1% had primary and secondary education, and .6% had no formal education. The marital status distribution was as follows: 58.9% single, 29.1% married, 6.8% in a common-law union, 4.5% divorced, and 0.6% widowed. According to the socioeconomic level categories proposed by the AMAI (2020), 43.7% corresponded to level A/B, 23.3% to C+, 20.1% to C, 8.7% to C-, 2.9% to D+, and 1.3% to D. The average reported weekly expenditure was $1,888.28 MN (with a standard deviation of $2,291.14 MN) (equivalent to an average of $113.14 US, with a standard deviation of $137.24 US).

All participants were invited to participate voluntarily without receiving any financial compensation. Their anonymity and the confidentiality of their data were assured. Informed consent was provided verbally and in writing to indicate their agreement. The study and consent procedures were performed in accordance with the ethical standards of the Declaration of Helsinki (World Medical Association, 2013), and the ethical guidelines of the university committee.

#### Procedure

##### Questionnaires

Drawing from the prior study, redundant semantic indicators were removed from the 461 indicators identified through the content analysis. Subsequently, 70 items were created based on the nine dimensions identified in the focus groups, with each dimension represented by 7 to 9 items. The questionnaires used a 7-point Likert-type scale, ranging from 1 to 7, allowing participants to indicate their level of agreement with each statement. This 7-point scale range has been shown to provide enhanced precision for capturing nuances in attitudes, compared to scales with fewer response options (Finstad, 2010; Taherdoost, 2019). Additionally, to ensure the cultural validity of the scale, the items were designed to incorporate terminology and expressions commonly used in the local context. In constructing the questionnaire, a balance was struck between simplicity and detail to promote participant understanding and encourage genuine engagement.

The survey also collected sociodemographic data, including participants’ age, gender, and socioeconomic status, aligned with the guidelines provided by the Mexican Association of Market Research Agencies (AMAI, 2024). The questionnaire was administered online, and participants were granted access through a unique link that could be accessed from their personal devices. The survey was distributed across various groups of employees and universities, specifically targeting those who met the eligibility criteria. Participants were informed about the purpose of the study, the confidential handling of their data, and their involvement was strictly voluntary. The research project adhered to all relevant ethical requirements and protocols in alignment with the General Health Regulations, particularly the guidelines governing studies involving human participants.

##### Data Analysis

Data were analysed using the psych (Revelle, 2021) and lavaan package (Rosseel, 2012) in *R* (R Core Team, 2022). Descriptive statistics and independent samples t-tests were first conducted on the scale items. Subsequently, exploratory factor analysis (EFA) was performed, along with an examination of Spearman correlations between the factors. Additionally, Cronbach’s alpha (α) and McDonald’s omega (ω) were calculated, with McDonald’s ω generally providing a more accurate reliability estimate than Cronbach’s α under most conditions (cf., Zinbarg et al., 2006).

To further establish the validity of the scales, confirmatory factor analysis was conducted using Diagonally Weighted Least Squares (DWLS) with a robust approach and a polychoric correlation matrix. DWLS was selected due to its ability to handle moderate violations of normality (Flora & Curran, 2004). The analysis assessed various model fit indices, including the comparative fit index (CFI), Tucker-Lewis Index (TLI), root mean square error of approximation (RMSEA) along with its 90% confidence intervals, and the standardised root mean square residual (SRMR). A multigroup analysis was performed to assess measurement invariance, where the data was divided based on biological sex. This analysis followed a step-by-step approach, examining configural, metric, and scalar invariance. Finally, sex differences were analysed using Mann-Whitney U tests.

### Results

#### Descriptive statistics and item discrimination

The total scores and quartiles of the scale were used to create a new variable, dividing the high and low scores of quartiles 1 and 3. Student’s t-tests for independent samples were used to analyse the discrimination of the items. Based on this analysis, five items were found to have failed to discriminate between high and low scores, and were therefore excluded from further analysis. The remaining items showed statistically significant differences at p < .001.

#### Exploratory Factor Analysis (EFA)

In order to obtain evidence of the configuration of the components of the purchasing premises, an EFA was performed. A dimension reduction was used using the maximum likelihood extraction method with varimax orthogonal rotation without specifying a predetermined number of factors. With this process, items with factor loadings less than .40 or items that loaded onto two or more factors with a difference of .20 or less in their factor loadings were eliminated. 19 items were removed through this procedure (e.g., *everything must be bought for children to prevent them from having deficiencies; People need to win over their partner by buying them a lot of things.).*

**Figure 1:**
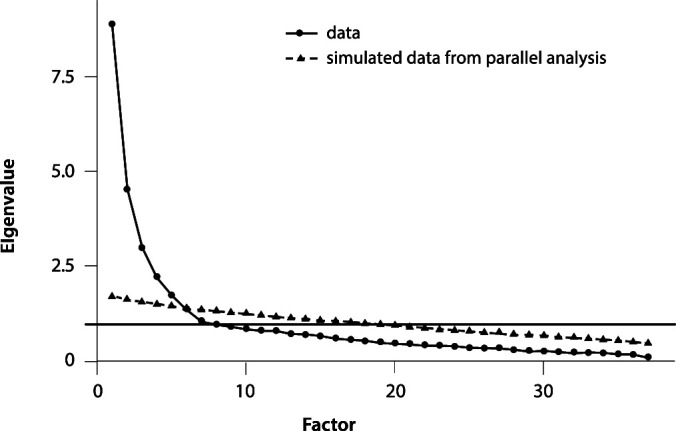
Exploratory Factor Analysis Scree Plot.

The analysis yielded a seven-factor solution with eigenvalues greater than 1 (see *Figure 1*), explaining 62.21% of the total variance (see *Table 2*). The factorial solution converged in six iterations and demonstrated a suitable Kaiser-Meyer-Olkin coefficient of .864 and a significant Bartlett’s test of sphericity (Bartlett = 5981.045, p < .001). Factor loadings greater than .409 and commonalities exceeding .323 were retained. Additionally, the internal consistency of the factors was examined through the calculation of Cronbach’s alpha and McDonald’s omega, with the composite reliability results generally ranging between .73 and .91 for all factors.

**Table 2:** Subscales, factor loadings, psychometric index of internal consistency, correlations between factors and descriptive statistics of the consumer socio-cultural premises scale.

Items		Subscales						
		F1 Affective Compensation	F2 Gender Stereotype	F3 Self–sacrifice	F4 Saving & Budgeting	F5 Family	F6 Social	F7 Hedonism
NP18*. You should earn your children’s affection by buying things for them*		**.914**	.197	.015	–.051	.081	.072	.102
NP16*. You should buy what your children want to prevent them from throwing tantrums*		**.843**	.252	.014	–.041	.073	.013	.159
NP17*. You should constantly purchase to demonstrate items for your your love family for them*		**.724**	.233	.107	.000	.090	.135	.239
NP19*. Family time should be compensated with gifts when there’s not enough to spend together*		**.703**	.242	.029	–.103	.035	.090	.115
NP20*. You should buy what you are asked to buy to avoid being seen as stingy*		**.662**	.217	.039	–.175	.029	.198	.137
NP21*. To compensate for emotional voids, people should fill them by making purchases*		**.592**	.213	.038	–.172	–.055	.132	.222
NP61*. Women should pay attention to the purchasing decisions made by men*		.230	**.732**	.007	–.056	.079	.081	.033
NP62*. A woman should prioritise the family when shopping and not focus on herself*		.124	**.687**	.095	.034	.245	.084	.067
NP60. *A woman should refrain from expressing her opinion while shopping*		.230	**.680**	–.028	–.015	–.029	.166	.061
NP68*. Men should prioritise family over personal indulgence when making purchases*		.083	**.651**	.101	.034	.257	.087	–.024
NP66*. Man should avoid giving opinions when making purchases*		.162	**.593**	.081	–.012	–.038	.110	–.005
NP67*. Men should pay attention to the purchasing decisions of women*		.122	**.593**	.082	.032	.084	.087	.074
NP 59*. Women should involve men in their shopping decisions*		.252	**.572**	.130	–.034	.171	.131	.083
NP65*. Men should ask women for their input when shopping*		.130	**.567**	.111	.083	.169	.103	.143
NP3*. If people want to buy something, prepared to they make should sacrifices to obtain it*		–.040	.085	**.844**	.046	–.029	.060	–.016
NP2*. People need to put in effort by working in order to afford the things they desire*		.050	–.037	**.779**	–.030	–.011	.063	.060
NP4*. You have got to give up some things to get stuff your family needs*		–.020	.165	**.745**	.075	.131	.135	–.066
NP5*. You should put an extra effort when you want to purchase a special treat*		–.034	–.003	**.686**	.095	.120	.043	.057
NP7*. People should exert extra effort to acquire the items they wish to purchase*		.154	.140	**.569**	–.085	.173	.039	.193
NP8*. People should seek extra income in order to purchase things they enjoy*		.031	.086	**.485**	.124	.188	–.045	.031
NP6. *You have got to go through tough times to get what your loved ones need*		.272	.245	**.485**	–.102	.140	–.011	.233
NP55. *People must be adapted to fit the budget and what you have*		–.072	–.094	.004	**.831**	.053	–.077	–.038
NP53*. People ought to spend in accordance with their dinancial capabilities*		–.197	–.092	.093	**.722**	.058	–.085	–.058
NP 54*. You should only spend what you've got*		–.082	.036	–.014	**.703**	.041	–.044	–.075
NP51*. People must settle with the budget they have*		.031	.115	.027	**.597**	.079	.012	–.047
NP52*. You must save to buy good products*		–.104	.024	.036	**.456**	.170	.058	–.028
NP37*. When shopping, a father should prioritise his family before himself*		.065	.224	.201	.035	**.671**	.014	–.018
NP38*. When shopping, a mother should prioritise her family over herself*		.070	.390	.146	.045	**.646**	.082	.104
NP34*. People should make purchases with the well-being of their family in mind*		.032	.134	.150	.239	**.492**	.146	.062
NP36*. People should consult with their family when making an purchase*		.019	.159	.034	.264	**.436**	.215	–.017
NP39*. People should spend on things that benefit the entire family*		.045	–.046	.195	.298	**.409**	.218	–.037
NP43*. You should ask a friend for their opinion when shopping*		.242	.262	.050	–.093	.076	**.778**	.076
NP45*. Purchases should be made in consultation with others*		.160	.245	.073	–.054	.219	**.675**	.087
NP44*. The opinion of older people should be consulted when making purchases*		.112	.236	.118	.026	.155	**.661**	.049
NP*happy* 14*. People must buy to feel*		.434	.090	.007	–.042	.004	.091	**.706**
NP 12*. People should buy to feel good*		.326	.213	.129	–.124	.017	.025	**.665**
NP13*. People should follow their heart when making a purchase*		.219	.029	.157	–.125	.037	.083	**.461**
	***Total***							
***Items number***	37	6	8	7	5	5	3	3
***% Explained variance***	62.21	24.11	12.08	8.25	6.09	4.69	3.65	3.30
***Cronbach’s Alpha***	.890	.919	.871	.856	.797	.754	.828	733
***McDonald’s Omega***	.900	.910	.861	.824	.780	.741	.828	743
**Interfactor correlations**		**F1 Affective Compensation**	**F2 Gender Stereo-type**	**F3 Self–sacrifice**	**F4 Saving & Budgeting**	**F5 Family**	**F6 Social**	**F7 Hedonism**
Gender Stereotype		.47^*^						
Self-sacrifice		.18^*^	.26^*^					
Saving & Budgeting		–.18^*^	.00	.04				
Family		.18^*^	.41^*^	.33^*^	.28^*^			
Social		.37^*^	.45^*^	.21^*^	–.06	.37^*^		
Hedonism		.55^*^	.28^*^	.23^*^	–.19^*^	.10	.26^*^	
***Mean***		1.50	2.51	4.23	5.61	4.31	2.60	2.50
***Standard deviation***		.95	1.30	1.39	1.13	1.30	1.40	1.36

^*^ p < .001

Factors related to saving and budgeting, family orientation, and self-sacrifice showed stronger agreement compared to a lower level of endorsement for factors associated with affective compensation and hedonism. Interfactor correlations indicated moderate to weak associations.

#### Confirmatory Factor Analysis (CFA)

**Figure 2:**
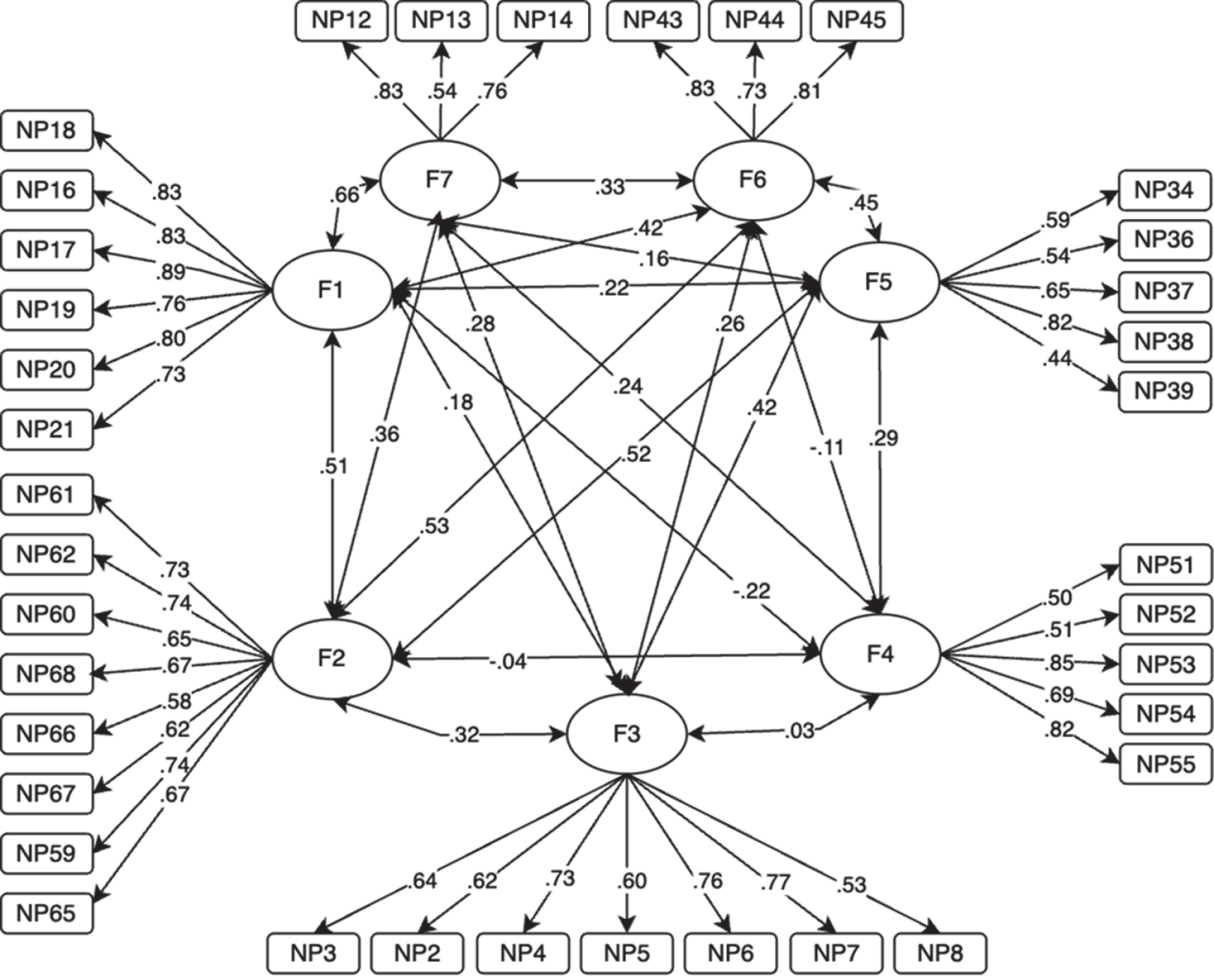
CFA Consumer Socio-Cultural Premises Scale

*Note: F1 = Affective Compensation; F2 = Gender Stereotype; F3 = Self-sacrifice; F4 = Saving & Budgeting; F5 = Family; F6 = Social; F7 = Hedonism.*

Confirmatory factor analysis using structural equation modelling with a diagonally weighted least squares estimator and robust standard errors was conducted to further examine the fit of the proposed seven-factor model and the data. Assessing measurement models through confirmatory factor analysis is crucial for evaluating the psychometric properties of a scale, as it allows researchers to evaluate how well the observed variables represent the underlying latent constructs. The CFA of the seven-factor model proposed in this study showed a somewhat adequate fit, with the following fit indices: χ^2^ = 942.18, df = 608, p < .001, CFI = .959, TLI = .955, RMSEA = .042 [90% CI 0.037 – .047] and SRMR = .072. Typically, CFI and TLI values exceeding .95 indicate an excellent model fit, while values between .90 and 0.95 suggest an acceptable fit. Excellent fit is also indicated by an SRMR value below .08 and an RMSEA below 0.6 (Bentler, 1990; Hu and Bentler, 1999; Schweizer, 2010; van de Schoot et al., 2012). The results of the chi-square test were provided, although this test statistic may not be reliable for larger samples (Byrne, 2001). Overall, the proposed seven-factor model showed an adequate fit to the data, indicating that this model is supported (see *Figure 2*).

#### Gender Measurement Invariance

Establishing measurement invariance across demographic groups ensures that observed differences in scale scores reflect true variations in the underlying construct, rather than differences in how the groups interpret the scale items. To assess this, a multigroup confirmatory factor analysis was conducted. This approach involves testing a series of nested models to assess the equality of factor loadings, intercepts, and residual variances across the different groups. By sequentially constraining these parameters to be equal across groups, researchers can determine whether the scale exhibits configural, metric, scalar, and residual invariance (Boateng et al., 2018). The results showed that the proposed seven-factor model of the scale exhibits measurement invariance between men and women (See *Table 3*).

**Table 3:** Testing for factorial invariance of the Consumer Socio-Cultural Premises Scale across women (n= 143) and men (n= 166) groups

Model	χ^2^	*df*	*CFI*	*RMSEA* (90% CI)	*SRMR*	Model Comparison	Δ*χ^2^*	Δ*df*	Δ*CFI*Δ	*RMSEA*	Δ*SRMR*
(M0)	1246.99	1216	.996	(.000 .013 – .025)	.083	–	–	–	–	–	–
(M1)	1319.31	1246	.991	(.000 020 – .029)	.086	M0 – M1	72.32	30	.005	.007	.003
(M2)	1340.05	1276	.992	(.000 .018 – .028)	.084	M1 – M2	20.74	30	.001	.002	.002
(M3)	1357.40	1313	.994	(.000 015 – .026)	.086	M2 – M3	17.35	37	.002	.003	.002

*^a^ M0 = configural, ^b^M1 = metric, ^c^M2 = scalar, ^d^M3 = residual.*

Cross-gender comparisons, conducted after establishing measurement invariance, revealed significant differences in the latent means of five out of the seven factors analysed. Men show higher scores than women in affective compensation and hedonism, while women show higher mean scores in self-sacrifice, saving and budgeting, and family (see *Table 4*). This suggests that although the underlying factor structure is equivalent for men and women, there are differences in the degree to which certain consumption premises are endorsed by each gender.

**Table 4:** Comparison of Consumer Socio-Cultural Premises Scale Factors between women and men

	Women	Men	Confidence Interval 95%	*W*	*p*-value	*Rank-Biseral Correlation*
	*M*	*M*				
	***(SD)***	***(SD)***				
AffectiveCompensation	1.43 (.91)	1.56 (.99)	[–.41, .21]	12912.50	.141	.08
Gender Stereotypes	(2.35 1.24)	(2.65 1.35)	[.01, .26]	13496.00	.037	.13
Self-sacrifice	(4.09 1.39)	(4.36 1.37)	[–.01, .24]	13309.50	.066	.12
Saving Budgeting &	(5.74 1.10)	(5.50 1.14)	[–.25, .01]	10346.50	.051	–.12
Family	(4.13 1.32)	(4.46 1.27)	[.02, .27]	13714.00	.018	.15
Social	(2.35 1.30)	(2.81 1.45)	[.05, .30]	13995.50	.006	.17
Hedonism	(2.46 1.43)	(2.53 1.30)	[–.07, .18]	12499.50	.417	.05

## Discussion

In accordance with the theoretical framework proposed by Díaz-Guerrero (1955, 1972, 1974) regarding socio-cultural premises, this research explores the norms associated with the acquisition of products and services. The identified categories provide insight into cultural propositions that span various social spheres, including family and personal realms like self-sacrifice, hedonism, and negative sentiments. They also encompass factors related to the product or service itself, such as payment methods, marketing, and the point of sale. These premises emphasise the significant influence of others and the roles inherent in familial and societal contexts, as well as gender dynamics, the impact of associations within an individual’s social sphere, and purchase behaviours driven by social acceptance, economic maximisation, and product evaluation based on criteria such as quality, functionality, and price. From a social identity perspective, the socio-cultural components identified by the Consumer Socio-Cultural Premises Scale closely reflect the fundamental aspects of social identity theory. This scale captures the significant in-group identities and values that shape consumers’ self-perception, influencing their attitudes and behaviours. Elements such as family, emotional compensation, gender stereotypes, and self-sacrifice illustrate the essential impact of social group affiliations and normative influences on consumer psychology. These findings enhance our understanding of social identity theory (Tajfel, 1978) by demonstrating how culturally specific socio-cultural principles serve as cognitive and motivational frameworks that shape consumer self-categorisation and comparisons of intergroup dynamics.

The findings align with elements previously identified in Mexican cultural studies, such as family orientation as a fundamental and most important element (Díaz-Guerrero, 1972; Díaz-Loving et al., 2011, 2015). Visible in the present study are traditional elements like the father’s supremacy in decision-making and the mother’s self-sacrifice when making purchases. Additionally, gender stereotypes emerged, such as men as providers, and couple dynamics in line with previous evidence (*e.g*., Díaz-Loving & Sánchez Aragón, 1998; Padilla Gámez & Díaz-Loving, 2013; Rocha-Sánchez & Díaz-Loving, 2005; Stauss, 2023) that in the present study, highlighted the role of purchases in the courtship process. Emotional factors were also evident, like using purchases to express love by buying everything for a loved one. Premises related to confronting stressful situations, reflecting the Mexican philosophy of life (Díaz-Guerrero, 1984), emerged, such as boldness and caution when choosing new products. Additionally, the study uncovered new factors, such as considerations regarding time recompense, societal embrace, familial tradition, and those pertaining to specific goods and services. These factors appear to enhance the framework of interaction protocols, observed not only among individuals but also between individuals and objects, evidence in line with previous research highlighting the importance of offering a framework for future work to delve into these differences and their underlying causes (Fonseca-Pedrero et al., 2011).

The scale has shown adequate psychometric properties across exploratory and confirmatory techniques. The findings of this study suggest that the Consumer Socio-Cultural Premises Scale may serve as a valuable tool for researchers interested in further exploring the socio-cultural factors that shape consumer behaviour, particularly within cultural contexts where interpersonal relationships and family dynamics are paramount. The establishment of measurement invariance in the scale ensures that even though the measured premises are sensitive to variables such as the sex of the participants (Díaz-Guerrero, 1972; Díaz-Loving et al., 2015), the differences found when comparing adherence to norms regarding the purchase of products and services between men and women will be equivalent in metrics and construct across both groups. Thus, when analysing differences between men and women regarding the dimensions of the scale, a contrast is evident regarding the degree of adherence to gender stereotypes. It is observed that premises associated with gender stereotypes exhibit a higher prevalence of indicators for women compared to men. Men tend to report indicators reflecting the stereotype of being providers. In contrast, women often express stereotypes about purchasing without male involvement, making purchases to stand out among other women (Dennis et al., 2018; Sullivan et al., 2022). The higher evocation of indicators by women in this context may stem from internalised social pressures regarding women’s roles within an inequitable system. In terms of self-sacrifice, it also exhibits a discernible gender disparity. Men tend to approach sacrifice through distinct and sporadic expenditures, such as “I found myself financially stretched... I ended up dining at a family restaurant, but at least I don’t have to worry about paying for electricity anymore.” Conversely, women frequently encounter situations that demand sacrifice, such as “One must be willing to incur debt in order to provide for their children,” illustrating a continual need for sacrifice. This distinction suggests disparate internalisation within the gender groups.

In the same line, social norms in purchases, those that refer to consulting with others when making purchases, the results seem to show that both men and women follow patterns of interdependence. This is contrary to the proposal that men have self-construals of independence and autonomy and women as being more interdependent (Cross & Madson, 1997). This is supporting evidence about interdependent conceptions in both men and women, where women consider the reference group based on closeness and relationship, while men’s considerations are based on the interaction of more collective aspects. (Gabriel & Gardner, 1999; Seeley et al., 2003). For instance, in the domain of social acceptance and status, it was observed that women expressed a significantly higher level of emphasis compared to men. An internal analysis revealed that this variance is attributed to the existence of internal pressures among women themselves. Expressions such as “comments are made in the restrooms if you carry a good quality handbag” and “there is a continuous competition among us to ascertain who possesses the finest handbag” were identified. This shows that women face ongoing societal pressure, not only culturally but also from within their own gender group. Social patterns have been identified even in situations of perceived trust, where men trust individuals more based on whether they share group members, in contrast to women, who trust those who share relationship connections (Maddux & Brewer, 2005). Thus, the social purchasing premises include friends, older people and acquaintances, which seem to be elements that are not totally relational for women. This would make them not significant to the same magnitude as men when adopting norms from said groups.

## Conclusion

The research presented in this study illustrates the socio-cultural factors involved in individual purchasing behaviour. It adopts an ethnopsychological approach to analyse consumer behaviour, capturing idiosyncratic elements relevant to purchasing dynamics. These findings align with prior research on Mexican family dynamics (Díaz-Guerrero, 1972; Díaz-Loving et al., 2011, 2015). A key challenge is to develop a measurement tool that captures both culturally specific purchasing beliefs and universal factors (Leung et al., 2002; Leung & Bond, 2004). The study builds on prior work, shedding light on beliefs related to managing economic challenges, such as boldness and caution in purchasing decisions, in line with the concepts investigated by Diaz-Guerrero (1984).

This underscores the need to continue developing instruments that can accurately identify and measure the impact of socio-cultural premises on diverse consumption behaviours. The scale presented here provides a novel instrument to analyse such relevant phenomena, which have received limited empirical examination despite their importance in understanding consumer behaviour in different contexts. Future research should explore the relationship between the identified premises and other relevant variables, such as economic factors, advertising exposure, media consumption, and psychological traits, to gain a more comprehensive understanding of their influence on consumer decision-making and behaviour.

## Limitations

The study presented here has some limitations. While the findings offer valuable insights into the impact of sociocultural factors on purchasing behaviour among Mexican consumers, it is important to expand the research to include other cultural contexts in order to validate the generalisability of the Consumer Socio-Cultural Premises Scale and gain a deeper understanding of how cultural influences shape consumer decision-making. Additionally, the cross-sectional design used in this study limits our ability to understand how these sociocultural premises may change over time, especially in response to broader societal shifts. Furthermore, relying solely on self-reported data introduces potential biases related to social desirability. Future studies could consider using alternative methodologies, such as observational techniques or longitudinal designs, for a more comprehensive understanding of this phenomenon. It’s also essential that further refinement and validation be carried out during scale development by incorporating feedback from field experts and conducting confirmatory factor analyses on independent samples.

## Future Research Directions

The development and validation of the Consumer Socio-Cultural Premises Scale opens up several avenues for future research. First, the scale should be tested in other cultural contexts to examine its cross-cultural applicability and measurement invariance. Second, researchers could explore how the socio-cultural premises captured by this scale interact with other individual difference variables (*e.g*., personality, values) to influence consumer attitudes and behaviours. Third, longitudinal studies could investigate how these socio-cultural premises evolve over time and impact consumer decision-making.

Additionally, future research could examine how the socio-cultural premises scale performs in predicting specific consumer outcomes like brand loyalty, sustainable consumption, and digital piracy. Incorporating the scale into more comprehensive models of consumer behaviour would also be a fruitful area for future inquiry.

## References

[ref1] AMAI. (2024). Niveles socioeconómicos de la Asociación Mexicana de Agencias de Inteligencia de Mercado y Opinión Pública AC [Socioeconomic levels of the Mexican Association of Market Intelligence and Public Opinion Agencies AC]. https://nse.amai.org/niveles-socio-economicos-amai/

[ref2] Appelhans, B.M., Tangney, C.C., French, S.A., Crane, M.M., & Wang, Y. (2019). Delay discounting and household food purchasing decisions: The SHoPPER study. Health Psychology, 38(4), 334–342. 10.1037/hea0000727 30896220 PMC6430149

[ref3] Asch, S.E. (1955). Opinions and social pressure. Scientific American, 193(5), 31–35.

[ref4] Bentler, P.M. (1990). Comparative fit indexes in structural models. Psychological Bulletin. 107(2), 238–246. 10.1037/0033-2909.107.2.238 2320703

[ref5] Betancourt, H., Flynn, P.M., Riggs, M., & Garberoglio, C. (2010). A cultural research approach to instrument development: the case of breast and cervical cancer screening among Latino and Anglo women. Health Education Research, 25(6), 991–1007. 10.1093/her/cyq052 20864605 PMC2974838

[ref6] Boateng, G.O., Neilands, T.B., Frongillo, E.A., Melgar-Quiñonez, H., & Young, S.L. (2018). Best practices for developing and validating scales for health, social, and behavioral research: A primer. Frontiers in Public Health, 6, 149. 10.3389/fpubh.2018.00149 29942800 PMC6004510

[ref7] Byrne, B.M. (2001). Structural equation modeling: perspectives on the present and the future. International Journal of Testing, 1, 327–334. 10.1080/15305058.2001.9669479

[ref8] Cialdini, R.B., & Trost, M.R. (1998). Social influence: Social norms, conformity and compliance. In Gilbert, D.T., Fiske S.T., & Lindzey G. (Eds.), The handbook of social psychology (pp. 151–192). McGraw-Hill.

[ref9] Cross, S.E., & Madson, L. (1997). Models of the self: Self-construals and gender. Psychological Bulletin, 122(1), 5–37. 10.1037/0033-2909.122.1.5 9204777

[ref10] Cruz del Castillo, C., Díaz-Loving, R., & Miranda Nieto, E. (2009). Construcción de una escala sobre normas y valores en universitarios mexicanos [Construction of a scale on norms and values in Mexican university students]. Interamerican Journal of Psychology, 43(2), 203–212.

[ref11] Dennis, C., Brakus, J.J., Ferrer, G.G., McIntyre, C., Alamanos, E., & King, T. (2018). A Cross-national study of evolutionary origins of gender shopping styles: she gatherer, he hunter? Journal of International Marketing, 26(4), 38-53. 10.1177/1069031X18805505

[ref12] Deshpandé, R., Hoyer, W.D., & Donthu, N. (1986). The Intensity of Ethnic Affiliation: A Study of the Sociology of Hispanic Consumption. Journal of Consumer Research 13(2), 214-220. 10.1086/209061

[ref13] Díaz-Guerrero, R. (1955). Neurosis and the Mexican family structure. American Journal of Psychiatry, 112(6), 411–417. 10.1176/ajp.112.6.411 13275587

[ref14] Díaz-Guerrero, R. (1972). Una escala factorial de premisas histórico-socioculturales de la familia mexicana [A factorial scale of historical-sociocultural premises of the Mexican family]. Revista Interamericana de Psicología, 6, 235–244.

[ref15] Díaz-Guerrero, R. (1974). La mujer y las premisas histórico-socioculturales de la familia mexicana [Women and the historical-sociocultural premises of the Mexican family]. Revista Latinoamericana de Psicología [Latin American Journal of Psychology], 6, 7–16.

[ref16] Díaz-Guerrero, R. (1984). El impacto de la cultura iberoamaricana tradicional y del stress economico sobre la salud mental y fisica: instrumentacion y potencial para la investigacion transcultural [The Impact of Traditional Ibero-American Culture and Economic Stress on Mental and Physical Health: Instrumentation and Potential for Cross-Cultural Research]. Revista Latinoamericana de Psicología [Latin American Journal of Psychology], 16(2), 167–211.

[ref17] Díaz-Loving, R., Rivera Aragón, S., Villanueva Orozco, G.B.T., & Cruz Martínez, L.M. (2011). Las premisas histórico-socioculturales de la familia mexicana: su exploración desde las creencias y las normas [The historical-sociocultural premises of the Mexican family: their exploration from beliefs and norms]. Revista Mexicana de Investigación En Psicología [Mexican Journal of Research in Psychology], 3(2), 128–142.

[ref18] Díaz-Loving, R., Saldívar, A., Armenta-Hurtarte, C., Reyes, N.E., López, F., Moreno, M., Romero, A., Hernández, J.E., Domínguez, M., Cruz, C., & Correa, F.E. (2015). Creencias y normas en México: Una actualización del estudio de las premisas psico-socio-culturales [Beliefs and norms in Mexico: An update on the study of psycho-socio-cultural premises]. Psykhe [Psyche], 24(2), 1–25. 10.7764/psykhe.24.2.880

[ref19] Díaz-Loving, R., & Sánchez Aragón, R. (1998). Inventario de premisas histórico-socio-culturales de la pareja mexicana [Inventory of historical-socio-cultural premises of the Mexican couple]. In Reyes Lagunes I., Díaz Loving R., & Rivera Aragón S. (Eds.), La Psicología Social en México [Social Psychology in Mexico] (pp. 129–136). AMEPSO.

[ref20] Escobar-Mota, G., & Sánchez-Aragón, R. (2013). Validación psicométrica de la Escala de Premisas Histórico Socio Culturales de la Monogamia (EPHSCM) [Psychometric validation of the Scale of Historical Socio-Cultural Premises of Monogamy]. Revista Costarricense de Psicología [Costa Rican Journal of Psychology], 12(2), 155–175.

[ref21] Fagundes, D. (2017). The Social Norms of Waiting in Line. Law & Social Inquiry, 42(4), 1179–1207. 10.1111/lsi.12256

[ref22] Finstad, K. (2010). Response interpolation and scale sensitivity: Evidence against 5-point scales. Journal of User Experience, 5(3), 104-110.

[ref23] Flora, D.B., & Curran, P.J. (2004). An empirical evaluation of alternative methods of estimation for confirmatory factor analysis with ordinal data. Psychological Methods, 9, 466-491. 10.1037/1082-989X.9.4.466 15598100 PMC3153362

[ref24] Flynn, P.M., Betancourt, H., & Ormseth, S.R. (2011). Culture, emotion, and cancer screening: An integrative framework for investigating health behavior. Annals of Behavioral Medicine, 42(1), 79–90. 10.1007/s12160-011-9267-z 21472484 PMC3584161

[ref25] Fonseca-Pedrero, E., Sierra-Baigrie, S., Giráldez, S.L., Paíno, M., & Muñiz, J. (2011). Dimensional structure and measurement invariance of the youth self-report across gender and age. Journal of Adolescent Health 50(2), 148-153. 10.1016/j.jadohealth.2011.05.011 22265110

[ref26] Gabriel, S., & Gardner, W.L. (1999). Are there “his” and “hers” types of interdependence? The implications of gender differences in collective versus relational interdependence for affect, behavior, and cognition. Journal of Personality and Social Psychology, 77(3), 642–655. 10.1037/0022-3514.77.3.642 10510513

[ref27] Helweg-Larsen, M., & Lomonaco, B.L. (2008). Queuing among U2 fans: Reactions to social norm violations. Journal of Applied Social Psychology, 9, 2378–2392. 10.1111/j.1559-1816.2008.00396.x

[ref28] Hofstede, G. (2001). Culture’s Consequences: Comparing Values, Behaviors, Institutions and Organizations Across Nations. SAGE Publications.

[ref29] Hu, L., & Bentler, P.M. (1999). Cutoff criteria for fit indexes in covariance structure analysis: conventional criteria versus new alternatives. Structural Equation Modeling: A Multidisciplinary Journal. 6(1), 1–55. 10.1080/10705519909540118

[ref30] Krippendorff, K. (1980). Content analysis: An introduction to its methodology. SAGE Publications.

[ref31] Leung, K., & Bond, M.H. (2004). Social axioms: A model for social beliefs in multicultural perspective. In Zanna M. P. (Ed.), Advances in Experimental Social Psychology (pp. 119–197). Elsevier Academic Press. 10.1016/S0065-2601(04)36003-X

[ref32] Leung, K., Bond, M.H., De Carrasquel, S.R., Muñoz, C., Hernández, M., Murakami, F., …, & Singelis, T.M. (2002). Social axioms: The search for universal dimensions of general beliefs about how the world functions. Journal of Cross-Cultural Psychology, 33(3), 286–302. 10.1177/0022022102033003005

[ref33] Maddux, W.W., & Brewer, M.B. (2005). Gender Differences in the Relational and Collective Bases for Trust. Group Processes & Intergroup Relations, 8(2), 159–171. 10.1177/1368430205051065

[ref34] McCort, D.J., & Malhotra, N.K. (1993). Culture and Consumer Behavior: Toward an Understanding of Cross-Cultural Consumer Behavior in International Marketing. Journal of International Consumer Marketing 6(2), 91-127. 10.1300/j046v06n02_07

[ref35] Moon, J., Chadee, D., & Tikoo, S. (2008). Culture, product type, and price influences on consumer purchase intention to buy personalized products online. Journal of Business Research, 61(1), 31–39. 10.1016/j.jbusres.2006.05.012

[ref36] Padilla Gámez, N., & Díaz-Loving, R. (2013). Premisas familiares y socioculturales del emparejamiento [Family and sociocultural premises of couple relationships]. Enseñanza e Investigación En Psicología [Teaching and Research in Psychology], 18(2), 249–262.

[ref37] R Core Team. (2022). R: a language and environment for statistical computing. R Foundation for Statistical Computing, Vienna, Austria.

[ref38] Rahman, S. ur, Chwiałkowska, A., Hussain, N., Bhatti, W.A., & Luomala, H. (2021). Cross-cultural perspective on sustainable consumption: implications for consumer motivations and promotion. Environment Development and Sustainability 25(2), 997–1016. 10.1007/s10668-021-02059-8

[ref39] Revelle, W. (2021). Psych: procedures for personality and psychological research, R package version 2.1.9. Available online at: https://CRAN.R-project.org/package=psych

[ref40] Ribeiro, R.E.M., Oliveira, P.H. de S., Moura, K.B. de, Abreu, C.R.S. de, Filho, C.A. de S.R., Monteiro, L.F.S., & Barbosa, D.J.B. de P. (2021). Factors that influence the purchasing behavior of the consumer of natural products. Independent Journal of Management & Production 12(4). 10.14807/ijmp.v12i4.1358

[ref41] Rocha-Sánchez, T.E., & Díaz-Loving, R. (2005). Cultura de género: La brecha ideológica entre hombres y mujeres [Gender Culture: The Ideological Gap Between Men and Women]. Anales de Psicología [Annals of Psychology], 21(1), 42–49.

[ref42] Rong, H., Li, H., Lian, Z., & Zheng, J. (2020). The effect of culture on consumption: A behavioral approach. Journal of Asian Economics 67, 101180. 10.1016/j.asieco.2020.101180

[ref43] Rosseel, Y. (2012). Lavaan: an R package for structural equation modeling. Journal of Statistical. Software, 48, 1–36. 10.18637/jss.v048.i02

[ref44] Sánchez-Aragón, R., & Díaz-Loving, R. (2009). Reglas y preceptos culturales de la expresión emocional en México: Su medición [Cultural Rules and Precepts of Emotional Expression in Mexico: Their Measurement]. Universitas Psychologica [University of Psychology], 8(3), 793–805.

[ref45] Schweizer, K. (2010). Some guidelines concerning the modeling of traits and abilities in test construction. European Journal of Psychological Assessment, 26, 1–2. 10.1027/1015-5759/a000001

[ref46] Seeley, E.A., Gardner, W. L., Pennington, G., & Gabriel, S. (2003). Circle of Friends or Members of a Group? Sex Differences in Relational and Collective Attachment to Groups. Group Processes & Intergroup Relations, 6(3), 251–263. 10.1177/13684302030063003

[ref47] Sherif, M. (1936). The Psychology of Social Norms. Harper.

[ref48] Stauss, B. (2023). Gifts in romantic relationships: What enhances and what weakens the relationship?. In B. Strauss (Ed.), Psychology of Gift-Giving (pp. 85-105). Springer. 10.1007/978-3-662-66393-6_7

[ref49] Sullivan J., Ciociolo A. & Moss-Racusin C.A. (2022). Establishing the content of gender stereotypes across development. PLoS ONE 17(7): e0263217. 10.1371/journal.pone.0263217 PMC9275684 35819934

[ref50] Taherdoost, H. (2019). What is the best response scale for survey and questionnaire design; Review of different lengths of rating scale / attitude, scale / likert scale. International Journal of Academic Research in Management, 8(1), 1-10.

[ref51] Tajfel, H. (1978). Differentiation between social groups: Studies in the social psychology of intergroup relations. American Press.

[ref52] Thφgersen, J. (1999). The ethical consumer. moral norms and packaging choice. Journal of Consumer Policy, 22(4), 439–460. 10.1023/A:1006225711603

[ref53] Van de Schoot, R., Lugtig, P., & Hox, J. (2012). A checklist for testing measurement invariance. European Journal of Developmental Psychology, 9(4), 486–492. 10.1080/17405629.2012.686740

[ref54] Venkatesan, M. (1966). Experimental study of consumer behavior conformity and independence. Journal of Marketing Research, 3(4), 384. 10.2307/3149855

[ref55] World Medical Association. (2013). World Medical Association Declaration of Helsinki: Ethical Principles for Medical Research Involving Human Subjects. JAMA, 310(20), 2191–2194. 10.1001/jama.2013.281053 24141714

[ref56] Zinbarg, R.E., Yovel, I., Revelle, W., and McDonald, R.P. (2006). Estimating generalizability to a latent variable common to all of a scale’s indicators: a comparison of estimators for ω_h_. Applied Psychological Measurement, 30(2), 121–144. http://doi.org./10.1177/0146621605278814

